# Food Poisoning

**Published:** 1900-04-07

**Authors:** 


### Food Poisoning.

The result of the action brought by Mr. and Mrs. Tilden Smith against the Inns of Court Hotel Company, and tried in the Queen's Bench Division of the High Court on Thursday and Friday last week, is calculated to add a new terror to the lives of all shareholders in such undertakings. It will be remembered that on July 17th, 1899, ninety-one ladies and gentlemen partook of the ordinary *table d'hôte* dinner of the hotel in question, most of them being inmates at the time, while a few, among whom Mr. and Mrs. Smith were included, were the guests of inmates. More than one-third of the whole number were attacked next day with serious illness, the symptoms commencing, as a rule, about eighteen hours after the meal; and of those attacked two gentlemen, Mr. Prosser and Mr. Bartlett, died. Mr. Smith, the plaintiff, was taken ill about noon on the following day when at his office, and returned home to find Mrs. Smith still worse than himself. The cases were described as gastro-enteritis, and appear to have been of the ordinary type of food poisoning; but the most careful possible investigation by the medical officer of the hotel, the medical officer of health of the district, the practitioners called in, and the analysts subsequently employed, failed to throw the smallest light, or to afford any room even for conjecture, upon the question as to what might be the offending dish or material. Nearly two-thirds of those who dined experienced no ill consequences, and many of these fortunate persons appear to have eaten their way steadily through the *menu*. There was no ascertainable difference between the feeding of those who suffered and of those who remained exempt, and it does not appear that any of the dishes excited adverse comment, or gave rise to any suspicion, during the progress of the dinner. The evidence of the chief servants of the company, the secretary and the *chef*, disclosed only two weak points in the armour of the defence, one being that the meat was stored in a refrigerator, which was itself kept in the kitchen, "a very hot room," and that some tinned apricots had been taken from a tin which had been opened on the previous day and set aside. Counsel for the plaintiffs drew from Dr. Lovett, the Medical Officer of Health for St. Giles's, the statement that he "did not think it a proper thing to leave fruit in opened tins," and the same witness replied to Mr. Justice Grantham that, "if meat were tainted and then iced, the taste and smell would be to a large extent destroyed." His Lordship dwelt both upon the admissions and upon this part of the medical evidence, and the jury found a verdict for the plaintiffs, with £40 for expenses and £100 for damages. There was a stay of execution, we presume with a view to an appeal; and it is evident that, if the decision in the first trial should be upheld, and if the other sufferers or their representatives receive compensation on a similar scale, the profits of the hotel company for the year are not unlikely to be seriously curtailed.

Sir Edward Clarke, for the defendants, submitted that there was no case, no negligence having been proved; but Mr. Justice Grantham held that there was evidence to go to the jury. So large a number of people had been poisoned, that the meat had probably been largely infected; and, if so, according to the medical testimony, the infection would "probably" be discoverable. It is on this question, of course, that the scientific interest of the trial mainly turns. It may safely be assumed, considering the time which elapsed between the ingestion of the meal and the appearance of the symptoms, that the latter were in all probability not to be ascribed to an actual, but only to a potentially poisonous property; in other words, it may be assumed that the injurious food did not contain ptomaines or other poisons in a state of activity, but only bacteria capable of producing poisons. That bacteria capable of producing poisons may exist in meat, whether fresh or preserved, is a fact familiar enough; but the really important question is whether the presence of such bacteria, even when ascertainable by laboratory methods, is either ascertainable, or capable of being reasonably suspected, from such an examination of food supplies as would be undertaken, as a matter of course, by a careful steward or manager. It is to be hoped, in the event of there being a new trial, that this point will be thoroughly threshed out by expert witnesses, for it is, to say the least, not entirely satisfactory that a company should be cast in heavy damages on the basis of nothing more substantial than an opinion that the presence of infective material might "probably" have been discovered. There was no suggestion as to the methods of examination by which this probability might have been translated into fact; and the verdict, so far as we can judge of it, appears to have been little more than a decision as to which, of two apparently innocent litigants, should bear the loss incidental to an unavoihable misfortune.

